# Achieving Operational Excellence Through Artificial Intelligence: Driving Forces and Barriers

**DOI:** 10.3389/fpsyg.2021.686624

**Published:** 2021-07-08

**Authors:** Muhammad Usman Tariq, Marc Poulin, Abdullah A. Abonamah

**Affiliations:** Abu Dhabi School of Management, Abu Dhabi, United Arab Emirates

**Keywords:** operational excellence, artificial intelligence, driving forces, barriers, artificial intelligence operations

## Abstract

This paper presents an in-depth literature review on the driving forces and barriers for achieving operational excellence through artificial intelligence (AI). Artificial intelligence is a technological concept spanning operational management, philosophy, humanities, statistics, mathematics, computer sciences, and social sciences. AI refers to machines mimicking human behavior in terms of cognitive functions. The evolution of new technological procedures and advancements in producing intelligence for machines creates a positive impact on decisions, operations, strategies, and management incorporated in the production process of goods and services. Businesses develop various methods and solutions to extract meaningful information, such as big data, automatic production capabilities, and systematization for business improvement. The progress in organizational competitiveness is apparent through improvements in firm’s decisions, resulting in increased operational efficiencies. Innovation with AI has enabled small businesses to reduce operating expenses and increase revenues. The focused literature review reveals the driving forces for achieving operational excellence through AI are improvement in computing abilities of machines, development of data-based AI, advancements in deep learning, cloud computing, data management, and integration of AI in operations. The barriers are mainly cultural constraints, fear of the unknown, lack of employee skills, and strategic planning for adopting AI. The current paper presents an analysis of articles focused on AI adoption in production and operations. We selected articles published between 2015 and 2020. Our study contributes to the literature reviews on operational excellence, artificial intelligence, driving forces for AI, and AI barriers in achieving operational excellence.

## Introduction

Artificial intelligence is a technological concept in operational management, philosophy, humanities, statistics, mathematics, computer sciences, and social sciences. Artificial intelligence aims to create computers or machines to carry out jobs that generally need human intelligence. The sub-discipline of artificial intelligence is machine learning, which directs to statistical learning. Machine learning aims to create algorithms that can automatically manage information in actual-time and enhancing experience without being unequivocally customized. Supply chains are experiencing advantages from investments and progressive interest in artificial intelligence technologies (Voronkova, 2019). The latest information systems, such as wireless technologies, the internet of things, affordable sensors, and cloud storage, act as the underpinning technologies of artificial intelligence. Today, it is quite feasible that business processes and value chains are connected within and across organizations. Organizations can use smart devices, mobile applications, and point of sale technologies to accumulate geographic, demographic, and behavioral customer data in real time that helps develop products and services. The applications can help improve business functions using robotics and automatic systems. They allow marketing to forecast and understand customer’s demands more precisely. There is an importance of responsiveness within supply chain procedures as consumers demand custom-made products and peculiar services expeditiously. Supply chains and worldwide production network systems are experiencing effective modifications in the progressive business environment. The evolution of new technological procedures and advancements in production intelligence is producing positive impact on decisions, operations, strategies, and management. Businesses develop various methods and solutions to extract important information, such as big data with automatic production capabilities, and systematization for business affiliation. It helps to recognize barriers in enhancing organizational performance, such as equipment management, defect identification, time reduction of the cycle, speculation of demand, bioinformatics, and human resource (Deivanathan, 2019). Innovation in product development and smart technologies allows production intelligence to use soft computing, advanced algorithms, decision technologies, and findings. That can be in different information systems for advanced production systems, advanced equipment control, engineering data analysis, enterprise resource planning (ERP), manufacturing execution system, and supply chain management to improve decision excellence and effectivity of management. Smart production has become the trend with the logical incorporation of decision technologies and artificial intelligence to adopt the latest information technology (Mühlroth and Grottke, 2020).

Operational excellence is a concept from the eighteenth century covering a subject’s productivity, labor division, and the free market. Any organization’s success depends on operational excellence as it relates to the organization’s functions serving consumers. This concept is at the first operating stage that is a short distance away from the resources involved in its functions but is crucially associated with its planning. The three important sections in operational excellence are effectiveness, right the first time, and efficiency of procedures (Mangla et al., 2020). The concept of operational excellence could entail adopting industrial tasks and theories, like Industry 4.0, Reverse Logistics, Lean Six Sigma, Business Procedure Reengineering, and the Internet of Things, which are enablers of acquiring accurate results. Improving organizational competitiveness is evident through firm’s proper decisions and represents working efficiency (Danaher, 2018). Improvements and development resulting from operational excellence relate to revolution, the latest technologies, and solutions. Innovation typically permits small business operating expenses and increased revenues (Carvalho et al., 2019). Acquiring operational excellence relates to adopting individual management theories and techniques that grant an adequate cost level to be justified and innovativeness, leading to investments and persistent enhancement procedures through problem-solving techniques (Jamshidieini et al., 2017).

There are four drivers at the foundation of attaining operational excellence. The first one relates to the organization’s vision that explains the requirements. The engagement of people in the strategy implementation is the second driver. The third driver involves creating the proper process performance metrics to support the organizational strategy. The last driver is the technology used to support the required processes.

Advanced algorithms are one important type of technology service as a driver for operational excellence. The development of an advanced algorithm helps forecast customer needs. If an algorithm is available, competitors may eventually obtain it or develop a similar tool to maintain their competitive position. The firms that do not adopt similar algorithms will be at a great disadvantage and may lose their market value. Advanced algorithms are essential for companies to staying in business within highly competitive markets. Integrating big data technologies enables the evolution of productive algorithms to understand the customer needs, which can be exceptionally beneficial for decision makers (Shehadeh et al., 2016).

**Figure 1:**
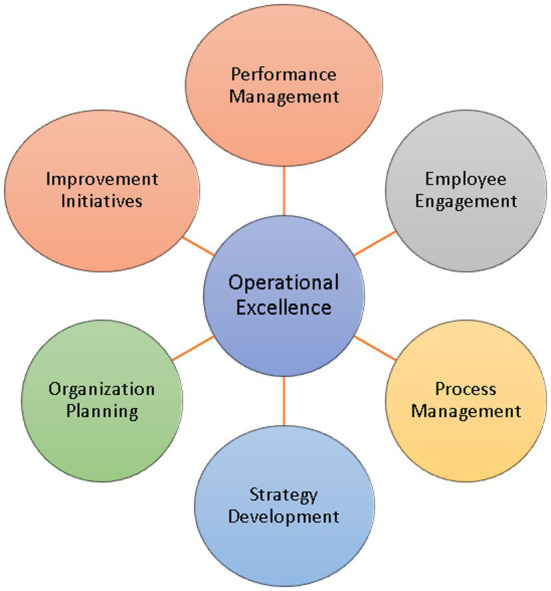
Operational excellence core functionalities.

A framework of the core functionalities for operational excellence is proposed in Figure 1. The figure depicts a better understanding of the role of artificial intelligence in operational excellence. Different operational excellence sections are distributed among the core functionalities, including performance management, employee engagement, process management, strategy development, organizational planning, and improvement initiatives. Artificial intelligence enhances the core functionalities of operational excellence.

**Figure 2:**
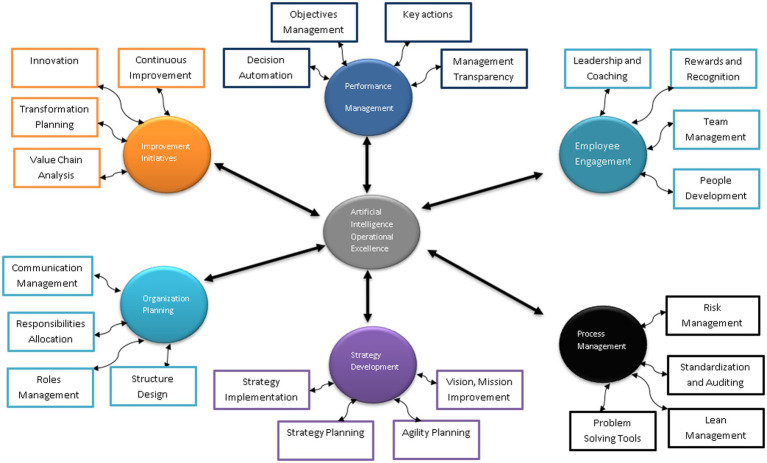
Artificial intelligence-based operational excellence framework.

The framework of artificial intelligence with operational excellence core functionalities is proposed in Figure 2. Artificial intelligence reshapes operational excellence by using different core functionalities to improve organization operations. Different automated intelligent algorithms find the patterns among the operations excellence’s different functions to automatically process the information. The outcome of artificial intelligence processing and real-time data can improve the organization’s operational decisions for achieving excellence. Artificial intelligence seeks to automatically select the right operational path connecting the various process within a business.

The current study focuses on the following research questions:

1. What is the connection between operational excellence and artificial intelligence?
2. What are the driving forces for achieving organizational performance by using artificial intelligence?
3. What are the barriers to achieving organizational performance using artificial intelligence?

This paper comprises six sections. The second section investigates the literature about artificial intelligence, operational excellence, and driving forces and barriers. In the third section, there is an explanation of the methodology used to perform this research. Outcomes and interpretations of this study are in the fourth and fifth sections, respectively. In the sixth section, we present the discussion, conclusion, and future research directions.

## Literature Analysis

Information technology is constantly growing with its application in various areas, including the educational field, healthcare field, or human resource field. Artificial intelligence and virtual reality are branches of computer science and are significant tools for improving human life or sustaining lifetime learning procedures (Stanica et al., 2018). Technology plays a vital role in influencing the social, political, cultural, educational, and organizational sectors in this rapidly changing world. The advancements in technology have made their importance valuable for all the sectors, subsequently increasing productivity by practical training and learning methods (Abduljabbar et al., 2019; Karsenti, 2019). Artificial intelligence is embedded in a computer or device by programming software, which helps to perform complex and specific tasks that were previously possible only with human intelligence. Handling complexity is the key factor while adopting artificial intelligence to solve complex problems. Despite the complexity and being time-consuming, artificial intelligence can perform various jobs in seconds without human’s assistance (Becker, 2017; Arrieta et al., 2020). Artificial intelligence is a technology that enhances the daily activities of social and economic life. It positively influences economic growth by solving various social obstacles. Artificial intelligence has recently magnetized the attention of many developed and developing countries, such as the United States, Europe, India, and China. The major focus is on the development of robotic technology and intelligence information technology. Even though the latest artificial intelligence technology is proving its excellence in obtaining specific models with various barriers, many intelligence information technology models require a self-idea function, rely on big data, and are complex (Skurdauskaitė, 2020).

### Adoption of Artificial Intelligence in Organizations

Artificial intelligence have shown to be useful in fulfilling the following requirements (Davenport and Ronanki, 2018).

#### Automation of Business Procedures

Digitization is captivating and modifies the market in the business sector. Manual workflows depending on paperwork lower firms’ manufacturing efforts (Scheer, 2017). The business should organize its internal procedures in an excellent way (Paschek et al., 2017). “Robotic Process Automation” tends to be the software-based solution to regulate business procedures that include daily tasks, systemized data, and determining results (Aguirre and Rodriguez, 2017). Involving robotics within manufacturing is not recent, but their functions and abilities have advanced significantly, surpassing human skills in many circumstances.

#### Obtaining Insight Through Data Analytics

Information plays a significant role in the decision-making procedures on the operational, strategic, and tactical stages. However, the calculation and accumulation of data within enterprises are rising quickly. Essentially, big data analytics is the use of the latest statistics applied to any type of electronic communication, which may include “messages, updates, images posted to social networks, readings from sensors, and GPS signals from cell phones” (Wamba et al., 2018; Allam and Dhunny, 2019). Big data analytics allows to advance the way large quantity of data are processed.

#### Rational Customer Engagement

With the rapid advancements in the technological fields, businesses are maintaining personal connections with customers, and brands are gradually pursuing to maintain a connection with the customers on the digital mediums. There is a growth of a broad range of communication procedures on several platforms, such as consumer feedback, sharing videos of the brands on social media platforms, and creating blogs (Eigenraam et al., 2018). With the development in technology and digitization, social media platforms act as a medium to spread the information about the products in both businesses to business and business to consumer organizations (Pansari and Kumar, 2017; Choi et al., 2018).

#### Smart Agents

Many customers enjoy group-based online shopping. Smart agents based on advanced algorithms can negotiate to lessen the efforts while collecting the buyer’s information, transaction costs, and sellers’ negotiation. Smart agents can help other models other than C2B when there is a negotiation between buyers and sellers (Liang et al., 2019).

#### Recommendations About Products and Services

With the emergence of artificial intelligence, there is an evolution in product and service recommendation systems for organizations to increase sales, personalization, and engagement using easy-to-understand images and languages. With the rapid advancements in artificial intelligence, there is a development of concepts and priorities to enhance sales management (Singh et al., 2019).

#### Employee Engagement

Artificial intelligence can be used to improve employee management in two ways. First, firms can have easy access to a large amount of data related to their business functions to help achieve an effective decision-making procedure. Secondly, artificial intelligence’s constant evolution allows organizations to manage and process the data in real time (Robert et al., 2020).

#### Benefits for Employees

AI can impact employee’s physical and emotional engagement. It can provide a direct influence on employee benefits and improve organizational excellence (Alvi et al., 2020). For instance, in this technological era, online survey systems supported by AI can help recognize employee’s needs regarding their organization (Kang et al., 2016).

#### Human Resource Strategies

In this technological era, the human resource information system has a vital role in the decision-making procedure for effective human resource management. A semi-structured and unstructured process of HR decisions is attainable by adopting an intelligent decision support system (IDSS) with the combination of the knowledge discovery database (KDD; Masum et al., 2018).

#### Safety and Quality Analysis

Safety and quality are the main concerns for governments and the automobile sector. The main technical problems involve validating inductive learning in the modern environment inputs and attaining good levels of reliability needed for complete fleet formation. Moreover, the significant challenge may be creating an end-to-end structure and formation procedure that enhances the safety concerns limitless technical specialties into a combined approach (Koopman and Wagner, 2017).

### Operational Excellence

Operational excellence is a concept that focuses on problem-solving techniques and leadership skills as the main factor for continuous development. Firms are usually unsure how to proceed with operational excellence, and most organizations find it too broad or doubtful as it is a complicated concept to explain. The employee’s and manager’s attitudes are not simply a set of activities that organizations perform (Gólcher-Barguil et al., 2019).

### Continuous Improvement vs. Operational Excellence

Continuous improvement is the ongoing attempt to enhance organizational procedures, services, and products. It often occurs gradually instead of occurring instantly through some advanced innovation or constant progression. With continuous improvement, a firm has more chances to sustain and progress on these improvements as processes are built in to assure continuity (Sánchez-Ruiz et al., 2019). Although continuous improvement is significant, it is not sufficient on its own to maximize a firm’s improvement.

As a firm continues to clarify its procedures, services, and products, it requires a way to pursue progress (Benzaid and Taleb, 2020; van Assen, 2020). Operational excellence plays a vital part in this stage. Operational excellence is an outlook that accepts some regulations and tools to develop long-lasting improvement within a firm. Firms can achieve operational excellence when every individual in the firm can observe its value (Çaliş and Bulkan, 2015). Firms should diligently attempt to implement changes to seek the observed value. Essentially, operational excellence focuses on eliminating costs or enhancing firm’s productivity. It is about developing an organizational culture that will allow the firms to manufacture valuable products and services for the consumers and attain sustainable progress. Operational excellence is a concept that includes adopting appropriate techniques for the right procedures. When this occurs perfectly, the absolute working environment prospers, motivates, and empowers the employees (Sehnem et al., 2019).

### Principles of Operational Excellence

#### Respect Every Person

Every individual is valuable and has potential, so an employee deserves respect. The perfect way to exhibit with respect to the employees and organizations must involve them in requisite activities. It will positively enhance employee’s motivational levels to feel more empowered to present their ideas (Sony, 2019).

#### Lead With Modesty

Modesty includes a desire to listen and accept every individual’s suggestion, setting aside that individual’s position or status within the organization. Leaders should always lead with modesty (Sony, 2019).

#### Seek Excellence

Managers must try to simplify the working procedures without compromising on quality. Managers and employees should look for sustainable solutions when any problem arises. It increases perfection that is one of the competitive advantages (Sony, 2019; Dogru and Keskin, 2020).

#### Accept Scientific Ideas

Continuous thinking and experimentation lead to innovation. It is essential to explore and encourage new ideas without fear of defeat (Sony, 2019).

#### Focus on the Procedure

When there is a negative outcome from any procedure, managers often blame employees for it. However, the source of the negative outcomes is often because of faults in the design of a procedure. Even the best employees cannot continuously provide exceptional outcomes with flawed procedures. So, instead of blaming employees, it is imperative to obtain an accurate picture of the real cause and make the proper adjustments to achieve the essential outcomes (Sony, 2019).

#### Ensure Quality

If monitoring is done on every part of a procedure, there is a possibility of achieving high quality. It helps to arrange work areas in a way that will make it possible to become visible. When there is a problem at any stage, it is important to pause the working procedure to solve it (Gólcher-Barguil et al., 2019).

#### Pull and Flow Value

Every firm’s goal is to furnish the utmost value to its consumers. For this purpose, firms should ensure that the procedure and workflow are uninterrupted as disruption cause inefficiencies and waste. There is an importance of evaluating and integrating customer requirements in procedures to ensure a firm fulfills the proper requirements (Chiarini and Kumar, 2020).

#### Think Logically

There is an interconnection between all the parts working together. It is significant to recognize the connection between all the parts because it will allow them to make decisions. Organizations should avoid a localized vision and eliminate data flow and ideas (Postavaru et al., 2019).

#### Develop Purpose Constancy

Employees should understand the mission statement and objectives of the firm from day one. Firms should focus on these objectives continuously. Employees should be aware of the changes and goals and seek to achieve those goals. Understanding this will allow the employees to adjust their activities, objectives, and behavior to benefit the organization (Ivanov and Sokolov, 2019).

#### Value Creation for Customers

Firms must work to recognize the demands and expectations of their consumers. A firm that cannot create and deliver value to its customer does not remain sustainable (Heinonen et al., 2019).

### Operational Excellence Methods

Firms can enhance their performance and culture through operational excellence, helping in sustainable progress. Organizations should observe traditional events and look forward to a sustainable change system. Several popular methodologies for achieving operational excellence (Chakraborty et al., 2020) follow:

#### Lean Manufacturing

This method concentrates on systematically reducing waste during production procedures. It recognizes that every procedure has some restrictions and stresses all the efforts for improvement on those restrictions are the fastest way to success. The basic principles of lean manufacturing concentrate on improving the quality of products and services, reducing anything that is not important, and eliminating overall costs (Jordan and Mitchell, 2015; Kamble et al., 2020). Conventional lean manufacturing recognizes seven areas of waste that are generally known as “seven deadly wastes.”

- **Overproduction:** It occurs when employees create something before its requirement. It is an unfavorable form of waste in the form of inventory, which often hides substantive issues.
- **Waiting:** When employees have to wait for the next manufacturing stage, there is no additional value. It can be very surprising and informative to investigate each production stage and calculate the actual time adding value to a product versus non-value-added time.
- **Transport:** It is a waste because the movement of incomplete or undone products does not add value to a product.
- **Motion:** This stage considers all extra movement that does not add value to a product. This often occurs due to poor working processes.
- **Over-processing:** It occurs when there is excess time on processing rather than producing according to customer’s requirements. It is one of the most challenging wastes to eliminate, especially when producing a low level of product variety.
- **Inventory:** This kind of waste can occur for several reasons, such as when supply is greater than demand, there are opportunities for quantity discounts from suppliers, and work-in-progress is created due to separation of the production processes, or smoothing of production levels. Although there are reasons to create inventory, the Lean philosophy still recognizes it as a waste.
- **Defects**: This obvious waste occurs when mistakes in production cause parts or products that cannot be used. Either time and resources are wasted to fix the part, or it must be thrown away. In any case, the defective part creates waste (Jarrahi, 2018; Alefari et al., 2020).

#### Six Sigma

It is a set of techniques and tools to enhance business procedures that help produce better services and products. Six Sigma’s objective is to enhance the consistency of customer’s experience by recognizing and reducing variation. A Six Sigma organization will seek to create not more than 3.4 defects for every million opportunities. The definition of defect is any product not meeting the standards accepted by customers. DMAIC implementation can help to build Six Sigma business. DMAIC stands for “define, measure, analyze, improve, and control.” Following are the steps for this procedure:

**◆ Define:** In the first step, the organization clearly defines the problem in order to fix it. After identifying the problem, the organization can develop a strategy and evaluate the accessible resources.
**◆ Measure:** Organizations need to measure all accessible information and investigate the current processes closely with the current procedure. Where is the requirement for improvement? What is functioning correctly?
**◆ Analyze:** After the organization’s measurement of data, they can analyze its findings and find the source of the problem.
**◆ Improve:** After analyzing the data, organizations need to develop solutions. Solutions are adopted on a low scale to examine the necessary modifications.
**◆ Control:** After implementing the new procedures, the organization must find a path to maintain the procedure. It is important to ensure continuous improvement to make the procedure effective (Niñerola et al., 2019).

#### Kaizen

In Japanese, Kaizen means “continuous improvement.” It helps to adopt positive, continuing changes in the work environment. Kaizen’s leading principles are that the enhanced procedure leads to positive outcomes, group work is significant for success, and some procedures need improvement. Firms adopt Kaizen to assist them in developing a continuous improvement culture. Employees work together to attain ongoing changes in the working environment. Small modifications will combine to develop major outcomes. This method does not certainly only focus on small changes. It also concentrates on all employees’ involvement to impact actual change. Kaizen focuses on the importance of continuous improvement. Organizations need to continuously make improvements. Kaizen helps increase employees’ efficiency, reduce costs, and enhance customer’s experience (Shan et al., 2016).

### Achieving Operational Excellence

The ultimate objective of organizations focusing on continuous improvement is operational excellence. Techniques and tools are a practical step to begin but are not enough to attain long-lasting change. There is often no difference of opinion between artificial intelligence and automation in normal discussions. However, both concepts are quite different from each other. Organizations who use them complementary can provide significant benefits for business productivity. With improved efficiency, organizations have room to reconfigure resources and progress. Like ERP, automation software will also help extract data for extensive comprehension, recognize the latest income flow to investigate, and assist organizations in accepting innovative technologies (Kibria et al., 2018). Achieving operational excellence enhances the efforts between employees and tools. Artificial intelligence performs tasks previously done by humans to improve their performance and productivity. Before adopting artificial intelligence and automation in business procedures, organizations need to understand their integration with advanced technology (Found et al., 2018). Skill improvement is the fundamental requirement of an advanced generation. Technology-based employees accept the integration of technological knowledge and strategic planning. In a competitive organizational environment, there is a need for daily improvement. It defines the basic concept of organizational excellence. Organizations are adopting artificial intelligence to develop automatic responses to information technology’s crucial functions. Automation procedure involves modifying the methods that both information technology specialists and business leaders apply in their daily activities (Hertz et al., 2018). By eliminating human work in fields where there is no necessity for human interaction, artificial intelligence can develop a foundation that improves processes. With the latest evaluation in artificial intelligence, organization of any size can automate their operational activities to develop fast machine-learning techniques (Kumar et al., 2018). It implies that advanced technology can detect and resolve issues as soon as they occur. These are the self-healing protocols of artificial intelligence that lead to improved information technology repair response time, reduced costs, networks, and business infrastructure with more reliability (Rusev and Salonitis, 2016). Many organizations are replacing the manual workforce with artificial intelligence and automation, permitting information technology specialists to spare more time on innovation instead of concentrating on daily problem solving from calculating and transferring large amounts of information while lessening actual-time RAM errors. CPU adjustments and controlling artificial intelligence probabilities are substantially limitless (Laird et al., 2017). Some latest algorithms can forecast problems before they occur, implementing a proactive strategy to discover suitable solutions. Solution based on artificial intelligence is modifying the business world continuously. The main advantages of self-hearing artificial intelligence solutions are the potential to lower the problem-solving time, discover the problems before they occur proactively, the critical ability for cost-reduction, and the potential to enhance customer’s experience. IBM is developing the latest technologies to help the organizations align their procedure, lower operating costs, and develop innovative business ideas (Lee et al., 2018; Thürer et al., 2018).

### Driving Forces for Using Artificial Intelligence for Achieving Organizational Excellence

Following are the driving forces for using artificial intelligence for achieving organizational performance:

#### Improvement in Computing Abilities of Machine

The power and speed of computers are improving with each passing day. Organizations can perform their daily activities using computers embedded with artificial intelligence for improving their performance (Chen et al., 2018).

#### Development of Data-Based Artificial Intelligence

The development from rule-based artificial intelligence to data-based artificial intelligence enables machine learning. Machines can learn and adopt procedures independently without if/then algorithms. It acts as a driving force for achieving operational excellence as machines continue the procedures with minimal human involvement (Li et al., 2017).

#### Advancements in Deep Learning

Deep learning allows machines to recognize and perceive the world in new manners. Traditionally, machines could not differentiate between similar products. Advanced technologies can extract the data and start performing functions independently. The term intelligent machines depicts this concept. From the organizational perspective, these technologies help the firms smartly detect the requirements and fulfill them (John et al., 2020).

#### Cloud Computing

Cloud computing also acts as a driving force that enables machines to communicate and complete specific tasks. Organizations must collect and process data in the present era, and cloud computing makes it easier and transparent for the procedures (Bolodurina and Parfenov, 2017).

#### Managing the Data

Artificial intelligence provides the opportunity to analyze, process, and act according to the data with exceptional speed levels. Data management is the basic requirement for embedding artificial intelligence in business procedures. Artificial intelligence allows us to analyze, manage, and process data according to requirements. Artificial intelligence permits enhanced storage capacities to manage and save data and information (Harrison et al., 2019).

### Barriers in the Adoption of Artificial Intelligence for Achieving Organizational Excellence

Following are the barriers in the adoption of artificial intelligence for achieving organizational excellence:

#### Cultural Constraints

This constraint relates to intransigence to change. Humans follow habit patterns. Once humans discover a methodology of performing tasks that is effective or efficient, they like to stick to it. It mainly requires some confidence in organizations to say that the disturbances and costs involved in modifying procedures or adopting new procedures will be worthy of bringing profitability (Tarafdar et al., 2019). Resistance can be simple as reluctance to manage over control, whether that is straight away to machines or employees who manage the technological framework that makes artificial intelligence possible (Makridakis, 2017). Mostly, this observes the requirement for artificial intelligence and insufficient recognition of its benefits (Yigitcanlar et al., 2020). Education can help to overcome this barrier. People need to understand how advanced technologies from natural language processing to cloud computing can enhance productivity and lessen costs. Once people become aware of it, they repeatedly engage themselves to enhance the potential for productive change with artificial intelligence’s adoption (Fountaine et al., 2019).

#### Fear

Fear is a natural and comprehensible response of humans. Fear about the unknown is the ancient and powerful emotion of humans. Still, there are enormous things that humans cannot imagine about how artificial intelligence will change our future. Fear can be subsided by understanding how new jobs will be created. Decision-making procedure by computer algorithms is indeed difficult to understand. It creates a fear that humans are losing control over their tasks and are no longer experts in their work (Mata et al., 2018). If machines carry out tasks more effectively and efficiently, there could be a decrease in the demand for jobs in certain areas. It can lead to two different situations: Machines fulfill primary requirements, and humans can pursue innovative and leisure activities. Second, the dependency on artificial intelligence can cause unemployment and social disturbance. To avoid the barriers, the solution is to turn over to technological grounds to enhance human’s work instead of replacing them (Ploder, 2019).

#### Lack of Skills

It is an actual and crucial issue for many organizations requiring the adoption of artificial intelligence and shifting to other data-based frameworks for automatic modification. When it comes to organizational performance and growth because of artificial intelligence, a barrier exists due to the lack of skills and technology specialists with the training and experience required to adopt the essential organizational change and infrastructure. Even though artificial intelligence has been applied for several years, it is only recently that this talent is in demand by the industrial sector (Lee et al., 2018). The massive progress in demand means that those with capabilities can demand higher salaries and promotional positions in the firms that appoint them. Furthermore, firms now understand the need for artificial intelligence and invest in implementing this technological (Moulin-Frier et al., 2017). Google and Facebook, for instance, are considered greatly advantages as businesses who have talent, considering other firms face fierce competition to hire new talent. However, there is a possibility that this barrier will be overcome by society closing the gap between demand and supply. With the need for skills, there is an opportunity for the talent to grow. Another resolution is enhancing skills among employees. With an increase in the number of artificial intelligence solutions accessible as a service progresses, there will be a lower need for thoroughly trained employees in conventional science to implement artificial intelligence solutions to many business issues. Thus, it will be more accessible and less difficult for growing internal employees in AI areas (Jarrahi, 2018).

### Lack of Strategic Planning to Artificial Intelligence Adoption

Somehow or another, this is a blend of a few different obstructions – shortage of skills, cultural barriers, difficulty in management to understand the benefits and productivity of artificial intelligence, and technological transformation. According to the overall progress and development programs, the outcome is that artificial intelligence capabilities are not according to plans at a strategic level. The outcomes cannot address organizational benefits, overall progress, and development programs. The main reason is that when organizations gain awareness about the significance of adoption of artificial intelligence technology and the benefits it can provide, they are unable to consider it from a strategic point of view, or from a complete know-how about the goals and objectives of all characteristics of the operations of artificial intelligence, from data accumulation to uncovering the insights (Nguyen et al., 2019). The solution to this barrier is that firms must always assure a clear strategic plan before money and time spent on resource-intensive artificial intelligence ideas. They must have a clear understanding of the advantages they can bring forward. Companies should ensure their artificial intelligence is wholly associated with the business objectives and excellence, where every shareholder has complete knowledge about failure and success (Olsen and Tomlin, 2020; Raj et al., 2020).

## Methodology

**Figure 3:**
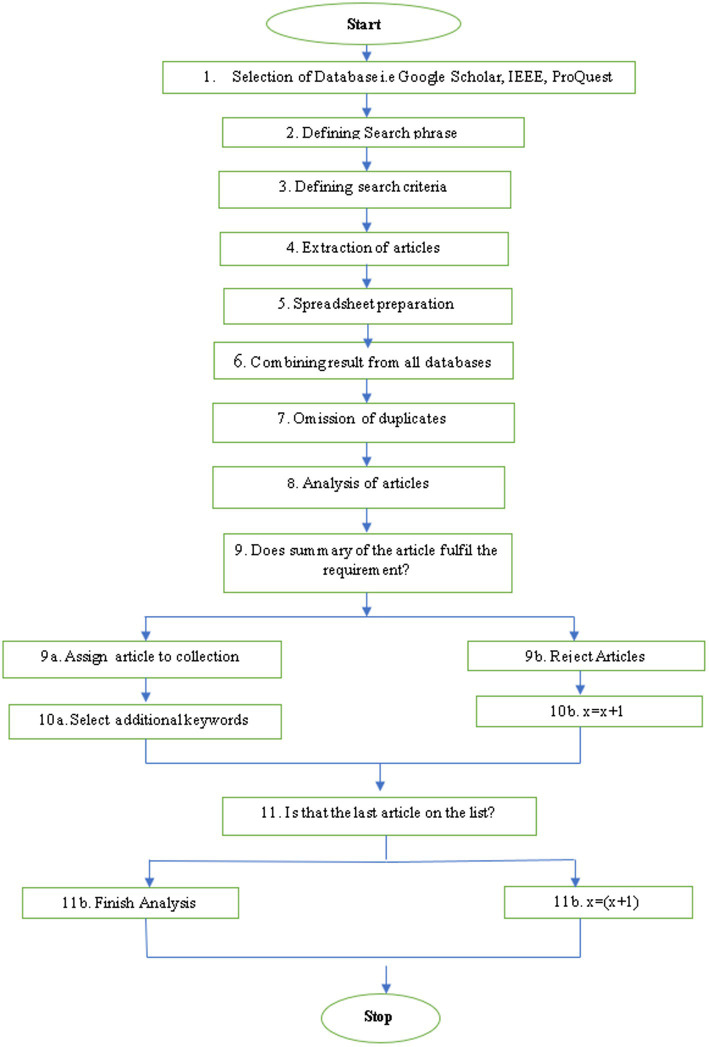
Article selection methodology.

The present paper conducts an in-depth systematic literature review on the intersection of AI and operational excellence. This method helps to provide a theoretical background for the study. The literature review should be a “systematic, explicit, and reproducible method for identifying, evaluating, and synthesizing the existing body of completed and recorded work produced by researchers.” Conducting a systematic literature review is known as “Fundamental Scientific Activity.” It also permits us to understand the research scope, present and learn about the previous literature, and discover the specific topic. We focused on English-language articles by following standard research procedures. The first step was a manual search of different relevant articles from EBSCO, ProQuest, Emerald Insight, Science direct, Taylor and Francis, Wiley, JSTOR, and IEEE. The methodology was designed in the following stages (Figure 3).

The articles were searched according to the search phrases and extracted from different databases. Initially, 1,854 articles were identified based on the generic keywords of artificial intelligence and operations excellence from 2015 to 2020. All results were stored and combined from all databases. Duplicate articles were omitted, leading to a net number of 850 articles compared according to the first English criteria. Articles were then documented in a “results” spreadsheet. An additional search was performed to ensure that all articles were found in the database to complete the process. Articles related to artificial intelligence and operational excellence were used in combination strings. Articles related to artificial intelligence and operations management were searched in the first set. The second set included articles about artificial intelligence, operations, operations research, operations excellence, and operations management. The strings were modified based on the different database types. The articles were based on quality and were filtered to only those in peer-reviewed journals and conferences, which led to a reduction of 350 articles. All articles were re-reviewed to ensure that they match the area of artificial intelligence and operations excellence. Abstracts of different articles were reviewed to match the study area further. That final filtering shortlisted the articles to 53, which were compared and discussed for in-depth analysis. The results are given in Table 1, which describes the stats on articles during the initial database search and after article processing. The first number in each column represents the initial search, and the second number represents the quantity after filtering. It is shown in Initial Search and After Processing format.

**Table 1:** Database search and Processing results.

	EBSCO	ProQuest	Emerald Insight	Science Direct	Taylor & Francis	Wiley	JSTOR	IEEE	Total
	Artificial Intelligence								
Operations	51.2	48.0	62.1	35.1	23.0	43.0	21.0	18.0	301.4
Operations excellence	22.3	29.1	33.1	23.0	18.1	28.1	13.1	11.2	177.10
Operations management	31.0	32.0	28.1	19.1	22.0	22.0	14.0	5.2	173.4
Operations risk	19.0	9.0	19.0	7.1	6.1	12.0	9.0	6.0	87.2
Operations research	11.0	9.1	23.0	6.1	7.1	11.0	19.0	8.0	94.3
Operations model	32.1	19.1	22.0	9.0	6.0	15.1	11.0	3.0	107.3
Operations framework	15.1	5.1	14.1	11.1	5.0	12.1	9.1	7.0	78.6
Organization and operations	45.0	9.1	18.0	3.0	4.0	12.0	13.1	4.0	108.2
Operations and structure	23.0	9.0	18.0	7.0	3.0	12.0	7.1	3.0	82.1
Operations improvement	22.1	15.0	11.1	12.1	11.1	16.0	9.0	6.0	102.4
Operations failure	18.0	9.1	16.0	7.0	5.1	25.1	12.0	8.0	100.3
Operations standards	12.0	8.1	9.1	9.0	3.1	9.0	14.0	3.0	67.3
Operations strategy	21.0	18.0	18.1	8.0	2.0	13.0	21.0	3.0	104.1
Operations planning	22.1	21.0	21.1	11.1	2.1	17.0	11.0	6.0	111.4
Operation decisions	15.0	17.1	16.0	4.0	2.0	21.1	19.0	9.0	103.2
Operation teams	12.0	8.1	7.0	3.0	1.0	9.0	11.0	3.0	54.1
Total	371.8	255.10	335.8	174.7	120.7	277.5	213.4	105.4	1850.53

## Findings and Results

**Figure 4:**
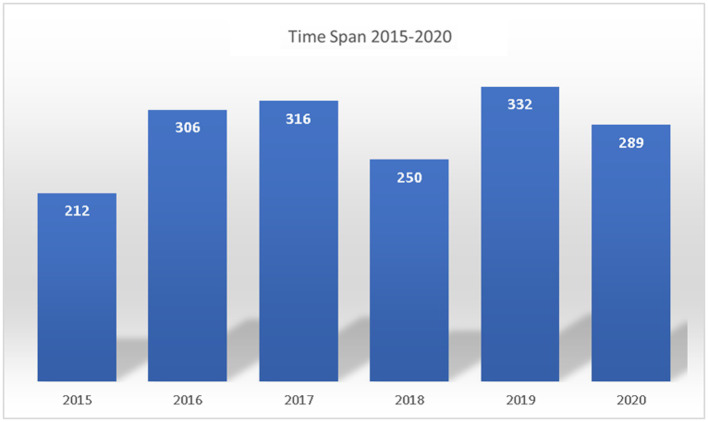
Time span for identified articles.

**Figure 5:**
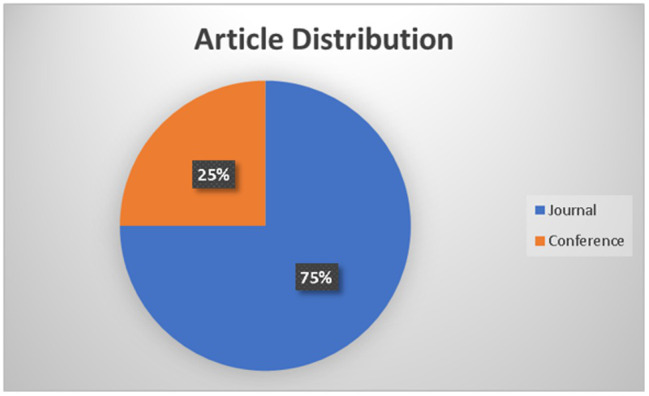
Article distribution.

The previous studies analysis proved that the artificial intelligence and operations excellence terms combined with other keywords identified a relatable need for coordination and commitment in the operation excellence field. The study findings are presented with tabulations and statistics to summarize the reviewed literature and discuss the research questions. Figure 4 provides the article’s numbers over the selected period sourced from peer-reviewed conferences and journals. Figure 5 provides an overview of the distribution of articles from the database search. 75% of the literature review was from peer-reviewed journals and 25% from conferences. Table 2 summarizes the artificial intelligence methods relevant to the operational excellence finalized for the 53 articles.

**Table 2:** AI method usage with operational excellence.

Areas	Artificial intelligence methods/Frequency
Operations	Automation (5)
	Neural networks (3)
	Decision trees (4)
	Modeling (6)
	Clustering (3)
	Support vector (2)
	Natural language processing (3)
	Fuzzy logic (4)
Operations management	Automation (7)
	Expert systems (1)
	Neural networks (3)
	Decision trees (2)
	Modeling (3)
	Simulations (5)
	Natural language processing (1)
Operations excellence	Decision support system (6)
	Modeling (5)
	Simulations (4)
	Automated planning (2)
	Neural networks (3)
	Deep learning (2)
Operations frameworks	Expert systems (2)
	Neural networks (5)
	Decision trees (6)
	Decision support system (7)
Operations models	Agent-based system (4)
	Expert systems (3)
	Modeling (2)
	Neural networks (4)
	Decision support system (3)
Operations decisions	Simulations (5)
	Decision trees (4)
	Neural networks (3)
	Image processing (2)
	Fuzzy logic (2)
	Decision support system (4)
	Natural language processing (4)
	Deep learning (4)

**Figure 6:**
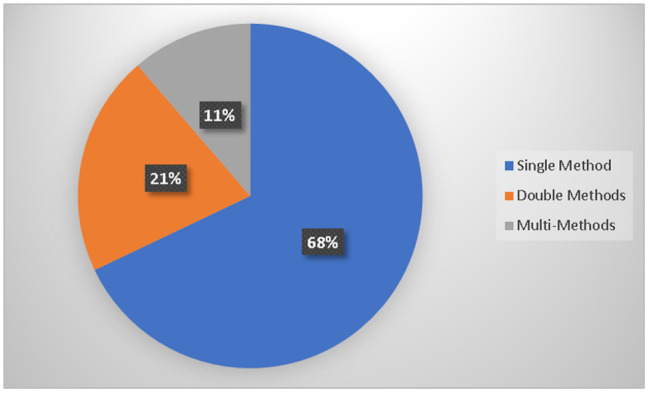
Summary of methods.

**Figure 7:**
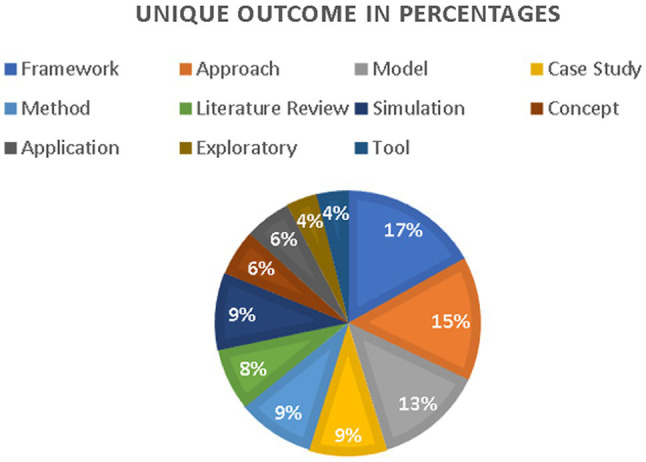
Unique outcomes in percentages.

Table 3 provides the frequency of AI methods used based on reviewed literature. Various AI methods were used in operational excellence based on the implementation. Most articles were based on using neural networks with a highest-ranking method with 21, decision support system as the second highest method of 20, decision trees and modeling as 16 each, simulation as 14, and automation as 12. The rest of the methods varies among different approaches. Figure 6 provides the overall summary of the methodologies used by the articles. 68% of the articles have used single methods, 21% have used double methods, and 11% have used multiple methods to solve operations problems for achieving excellence. Table 4 provides the frequency of the analyzed articles in terms of the unique outcome that shows the importance of using artificial intelligence for operational excellence. Figure 7 shows the summary of the articles reviewed in terms of a unique outcome. Most of the articles have used the framework as a unique outcome, nearly at 17%, the approach is being used as 15%, the model is being used as 13%, case study, and method as 9% each. The rest of the outcomes varies among the selection of outcomes by the researchers.

**Table 3:** Frequency of AI methods.

Method	Frequency
Neural networks	21
Decision support system	20
Decision trees	16
Modeling	16
Simulation	14
Automation	12
Natural language processing	8
Deep learning	6
Expert systems	6
Fuzzy logic	6
Agent-based system	4
Clustering	3
Support vector	2
Automated planning	2
Image processing	2

**Table 4:** Frequency of unique outcomes.

Unique Outcome	Frequency
Framework	9
Approach	8
Model	7
Case study	5
Method	5
Simulation	5
Literature review	4
Concept	3
Application	3
Exploratory	2
Tool	2

## Discussion

The results exhibit a connection between artificial intelligence and operational excellence that is proven by the selection of the 53 articles. It proves that 1 out of 12 articles about artificial intelligence refers to operational excellence. It also highlights that 12 percent of the 53 articles show a distinct connection between operational excellence and artificial intelligence. The results are significant because they depict that achieving operational excellence is dependent on the driving forces and resolving barriers. It is possible to achieve operational excellence by eliminating barriers. The analysis does not prove any specific methods to enhance operational excellence. It investigates the driving forces and barriers of using artificial intelligence to achieve operational excellence. The methodology allowed us to recognize how various keywords have a relationship and explain the research gaps.

### What Is the Connection Between Operational Excellence and Artificial Intelligence?

The answer to the first research question is we conducted the analysis that explains artificial intelligence and virtual reality are the branches of computer studies and are significant tools for improving human life or sustaining their lifetime learning procedures. Technology plays a vital role in influencing the social, political, cultural, educational, and organizational sectors (Siryani et al., 2017).

Intelligent agents can help models other than C2B when negotiating between buyers and sellers. With the rapid advancements in artificial intelligence, there is a development in many concepts and priorities to enhance product selling management. In this technological era, online survey systems help recognize employee’s needs regarding working in the organization (Wang et al., 2015). Operational excellence is a concept that focuses on problem-solving techniques and leadership skills as the main factor for continuous development. Firms are usually unsure how to proceed with operational excellence, and most organizations find it too broad or doubtful as it is a complicated concept to explain. The employee’s and manager’s attitude are not a set of activities that organizations perform. Firms can enhance their performance and culture through operational excellence, helping in sustainable progress. Organizations should observe past traditional events and look forward to a sustainable change system. Principles of operational excellence are: Respect every person, lead with modesty, seek excellence, accept scientific ideas, focus on the procedure, ensure quality, pull and flow value, think logically, develop purpose constancy, and value creation for customers.

### What Are the Driving Forces for Achieving Organizational Performance by Using Artificial Intelligence?

The second research question addresses the question that the driving forces for achieving operational excellence through artificial intelligence are improved machine computing abilities (Wirtz, 2019). Organizations can perform their daily activities using computers embedded with artificial intelligence to improve their performance. The second driver is the development of data-based artificial intelligence: Machines can learn and adopt procedures independently without the algorithm of if/then. The third driver is advancements in deep learning, which means that deep learning allows machines to recognize and perceive the world differently (Zhao et al., 2019). From the organizational perspective, these technologies help the firms smartly detect the requirements and fulfill them. The fourth barrier is cloud computing, which enables machines to communicate and work to complete specific tasks. In the present era, organizations have to collect and process data, and cloud computing makes it easier and transparent for the procedures. The fifth driver is managing the data. Artificial intelligence provides the opportunity to analyze, process, and act according to the data with exceptional speed levels. Artificial intelligence allows us to analyze, manage, and process data according to requirements.

### What Are the Barriers to Achieving Organizational Performance Using Artificial Intelligence?

The third research answer is the barriers in achieving organizational excellence through artificial intelligence that are cultural constraints; Once humans discover a methodology of performing tasks that is effective or efficient, they like to stick to it. It can be simple as reluctance to manage over control, whether that is straight away to machines or employees who manage the technological framework that makes artificial intelligence possible. Education can help to overcome this barrier. People need to understand how advanced technologies from natural language processing to cloud computing can enhance productivity and lessen costs. Once people become aware of it, they repeatedly engage themselves to enhance the potential for effective change with artificial intelligence’s adoption. The second barrier is fear. Fear is a natural and comprehensible response of humans. Fear about the unknown is human’s ancient and powerful emotion (Bottani et al., 2019).

For a quick understanding, the fear can circulate distance between the job and employees to get the salary. It creates a fear that humans are losing control over their tasks and are no longer experts in their work. To avoid this barrier, the solution is to turn over to technological grounds to enhance human work instead of replacing them. The third barrier is the lack of skills. It is an actual and crucial issue for many organizations requiring the adoption of artificial intelligence and shifting to other data-based frameworks for automatic modification (Huo et al., 2020). When it comes to organizational performance and growth because of artificial intelligence, a barrier exists due to the lack of skills and technology specialists with the training and experience required to adopt the fundamental organizational change and infrastructure (Gray-Hawkins and Lăzăroiu, 2020). Although there is a possibility that this barrier will be overcome by managing the demand and supply (Marcos et al., 2020), with the need for skills, there is an opportunity for the talent to grow. Another resolution is enhancing skills among employees. The fourth barrier is the lack of strategic planning for artificial intelligence adoption. Somehow or another, this is a blend of a few different obstructions – shortage of skills, cultural barriers, difficulty in management that affect the benefits and productivity of artificial intelligence, and technological transformation. The solution to this barrier is that pretty direct firms must always assure a clear strategic plan before money and time spent on resource-intensive artificial intelligence ideas without a clear understanding of the advantages they can bring forward.

## Research Implication

This study will help managers understand the barriers to adopting artificial intelligence to achieve operational excellence. This paper upgrades the previous understanding of operational excellence, artificial intelligence, and the drivers and barriers in adopting artificial intelligence. Knowing the drivers and barriers in adopting artificial intelligence for achieving operational excellence supports the organizations to maintain their competitive position. The focused literature review revealed that operational excellence could be implemented in a more efficient manner using artificial intelligence. The proposed framework for the operational excellence core functionalities can be extended according to organizational needs. Additionally, the in-depth framework for the artificial intelligence-based operational excellence framework provided the value-added areas that can help organizational leaders to focus on the essential domains as well-linked functionalities. Implementing the proposed frameworks will expedite the operational excellence in organizations with a clear roadmap to align the key performance indicators.

## Limitations and Directions for Future Research

There are some limitations to this study. First, the framework of this study focuses on prior literature. So, it is not practical to apply generalization. Furthermore, the study is restricted to operational excellence and artificial intelligence uses in the organization. Future researchers can investigate other factors that impact artificial intelligence’s adoption in other sectors, such as banking, construction, and health sectors. Additionally, the proposed frameworks can be used for implementation in an organization for a pilot run. The outcome of the results can be further verified through the pre- and post-implementation of the artificial intelligence-based operational excellence framework. Moreover, a case study-based approach can be undertaken to provide complete procedures and processes for other organizations and better understand artificial intelligence and operational excellence.

## Data Availability Statement

The original contributions presented in the study are included in the article/supplementary material, further inquiries can be directed to the corresponding author.

## Author Contributions

MT contributed to the ideation, conceptualization systematic analysis, review, collection of articles, visualization, and formatting of the articles. MP and AA contributed to the literature review, tabular result analysis, proofreading, logical flow, visualization enhancement, and final formatting. All authors contributed to the article and approved the submitted version.

### Conflict of Interest

The authors declare that the research was conducted in the absence of any commercial or financial relationships that could be construed as a potential conflict of interest.
